# Editorial: Acute-On-Chronic Liver Failure: Natural History, Mechanism, and Treatment

**DOI:** 10.3389/fmed.2022.867436

**Published:** 2022-03-11

**Authors:** Yu Shi, Yuchen Fan, Tao Chen

**Affiliations:** ^1^State Key Laboratory for Diagnosis and Treatment of Infectious, Collaborative Innovation Center for Diagnosis and Treatment of Infectious Disease, National Clinical Research Center for Infectious Diseases, The First Affiliated Hospital, Zhejiang University School of Medicine, Hangzhou, China; ^2^Department of Hepatology, Qilu Hospital of Shandong University, Jinan, China; ^3^Department and Institute of Infectious Disease, Tongji Hospital, Tongji Medical College, Huazhong University of Science and Technology, Wuhan, China

**Keywords:** acute-on chronic liver failure, natural history, mechanism, prognosis, diagnostic criteria

Acute-chronic liver failure (ACLF) is a lethal syndrome due to the acute exacerbation of underlying chronic liver diseases (CLDs), which is characterized by multi-organ failure with high short-term mortality. ACLF is one of the three leading causes of death in patients with chronic liver diseases. Although the concept of ACLF was proposed on 1995 (1), the study of ACLF has undergone explosive growth since the development of the EASL-CLIF criteria in 2013 (2). However, the controversy persists, particularly in the diagnostic criteria of ACLF, which mainly arises from the different backgrounds of underlying CLDs in the East and the West (3). In the West, alcoholic cirrhosis and bacterial infection constitute the major types of underlying CLDs and acute precipitating events in ACLF patients (4). Extrahepatic organ failures are frequent, manifesting in the first organ involved. In contrast, hepatitis B virus (HBV)-related ACLF is most common in China (5). Patients with HBV-ACLF usually have compensated liver cirrhosis or advanced CLDs. Liver and coagulation failure usually come first, followed by extrahepatic organ failure (6, 7). The proposed EASL-CLIF criteria is enlarging the interest in Eastern ACLF, leading to a plethora of publications. Therefore, the appearance of this special collection is very timely.

The first topic of the special collection is about ACLF's diagnostic criteria which is a fundamental issue and a focus of controversy in this research field. One of the critical questions is the threshold of organ failure for ACLF diagnosis, which varies among different criteria. The APASL consensus applies a serum bilirubin level ≥ 5 mg/dL and INR ≥ 1.5 to define severe liver dysfunction in patients with underlying CLDs (8). A higher threshold value of serum bilirubin ≥ 12 mg/dL and INR ≥ 2.5 is utilized by EASL-CLIF criteria to define liver and coagulation failure in patients with de-compensated cirrhosis (2). And a serum bilirubin ≥ 12 mg/dL and INR ≥ 1.5 is used to define ACLF in patients with HBV-CLDs by the COSSH criteria (6). In our special collection, two articles investigated the relationship between the continuous changes of serum bilirubin and INR, and 90-day mortality in patients with CLDs and those hospitalized for acute decompensation (AD) or acute liver injury. Qiao et al. found that a significant correlation between bilirubin and death began to be evident only when serum bilirubin exceeded 12 mg/dl in patients without cirrhosis, while any increase in bilirubin in patients with cirrhosis was associated with risk of death. Wang Y. et al. found that an INR of 1.6/1.7 was the starting point of coagulation dysfunction with a rapid increase in mortality in patients with cirrhosis or with advanced fibrosis. And a 28-day LT-free mortality of 15% was associated with an INR value of 2.1. In another study, Long et al. found that hepatic encephalopathy grades 3–4 were an independent risk factor for 28- and 90-day adverse outcomes in patients with acute-on-chronic liver diseases, and therefore could define brain failure in ACLF, regardless of the presence of cirrhosis. These three studies provide evidence for defining the threshold of organ failure for ACLF diagnosis and highlight that non-cirrhotic and cirrhotic ACLF may differ in the threshold of liver failure but share that of extrahepatic organ failure. On the other hand, in another article, Chen Y.-y. et al. extended the APASL criteria to patients with decompensated liver cirrhosis and identified a novel group of ACLF patients with short-term mortality similar to non-cirrhotic ACLF but higher long-term mortality (Xu et al.). Taken together, a basic question is raised by the four studies: do we require a unified diagnostic criteria or different criteria to identify patients with CLDs and high death risk?

ACLF is a lethal and highly dynamic disease. Liver transplantation can improve patient survival but is limited by the shortage of donor organs. The lack of other definite therapies puts the prediction of patients' prognosis in a very important position. Therefore, another Research Topic of this special collection is to develop a clinically useful tool to accurately predict the outcome of ACLF. Three articles reported their HBV-specific clinical prediction models, each was developed by multi-center data and verified by an external cohort with good discrimination and calibration (Chen J.-f. et al.; Yu et al.; Zhe-bin et al.). Importantly, a nomogram and online calculation tool was developed for the convenience of clinical use. Other articles explored the use of biomarkers, including testosterone and estradiol (Sun, Xu et al.), CD200R (Li Y. et al.), growth hormone (Wu D. et al.), neutrophil-to-lymphocyte ratio (NLR) (Liu et al.), and lymphocyte subsets (Li J. et al.), which may not only add to the performance of clinical prediction models, but also uncover some systemic features of ACLF in immunopathology and the endocrine system, and thereby provide clues for elucidating the pathophysiological mechanism. Another article developed a weighted score to predict the risk of invasive pulmonary aspergillosis (IPA), which is a lethal complication in patients with liver failure (Zhang et al.). However, all these clinical prediction scores and biomarkers should be tested further for their clinical utility in large cohorts and a real-world setting.

The third topic is to test novel therapies for this critical disease. One article reported encouraging results of a non-bioartificial liver support system (NB-ALSS) in treating patients with HBV-ACLF (Li J. et al.). The large, multi-center study enrolled 524 patients with HBV-ACLF and used paired analysis by propensity score matching to show that patients who received plasma exchange (PE)-based NB-ALSS had higher short-term and mid-term survival than those with standard medical therapy (SMT). Interestingly, further subgroup analysis revealed that PE-based NB-ALSS had the best efficacy in patients with ACLF grade 2 or a high MELD score of 30–40. The positive findings of NB-ALSS were in contrast with the futility of Western ACLF, despite of difference in devices. Another article performed a meta-analysis of the present literature on the use of granulocyte colony-stimulating factor (G-CSF) and demonstrated that G-CSF was not effective in the overall ACLF population. However, the subgroup analysis showed improved survival in Asian studies (Hou et al.). Although the findings of both articles are preliminary, a difference in pathophysiological mechanisms between Eastern and Western-defined ACLF can be speculated. For instance, there has been evidence that damage-associated molecular patterns (DAMPs) and pathogen-associated molecular patterns (PAMPs), while both involved in the development of systemic inflammation in ACLF, have differential contributions to the systemic innate immune derangement (9). Another important insight by the two articles lies in the potential presence of “efficacy threshold” and “treatment window” in treating ACLF, which indicates that risk stratification is critical in designing randomized controlled trials to test the efficacy of novel devices or drugs in the future. In addition to NB-ALSS, one article reported preliminary data of the efficacy of N-acetylcysteine in the treatment of HBV-ACLF, and its improvement of intrahepatic cholestasis and coagulation dysfunction of HBV-ACLF provides a new possibility for the treatment of HBV-ACLF (Wang M.-L. et al.). Another article studied the safety concern of the proton pump inhibitor (PPI) in ACLF (Sun, Ye et al.). The risk of PPI use in de-compensated cirrhosis has been debated, including the impact on risk of hepatic encephalopathy (HE), spontaneous bacterial peritonitis (SBP), and mortality. The article found that PPI use does not appear to increase mortality or the risk of HE and SBP in hospitalized de-compensated cirrhosis patients with and without ACLF.

The Research Topic also collected two up-to-date literature reviews. One summarizes the progress of systemic inflammation and immune cell paralysis and immunosuppression. The authors pointed out that the interaction between systemic inflammation and immunosuppression needs to be clarified. The review also provides insight into the specific precipitants of ACLF including HBV reactivation, extensive alcoholism, hepatitis E virus infection and flare-up of autoimmune hepatitis, complications including bleeding, hepatic encephalopathy (HE) and infections, and treatment options, in particular emerging stem-cell based therapies (Wu J. et al.). While the other review narrows on hepatitis E virus-related ACLF, mainly on immunological manifestations and mechanisms (Khanam and Kottilil).

Considering that ACLF is a highly complex syndrome in clinical phenotype and pathophysiological mechanisms, it is an apparent huge challenge to modify the clinical trajectory of this critical disease. Fortunately, broad interests on ACLF have been spiked both in the Eastern and Western scientific communities and many efforts have been put forward to establish diagnostic criteria, characterize natural history, and develop prediction tools and novel therapies. This Research Topic seeks to collect articles or reviews that provide either insights into the understanding of the disease or translational potential for better clinical care. Finally, we hope you enjoy reading this collection.

## Author Contributions

YS, YF, and TC conceived the idea. YS wrote the draft. YF and TC reviewed and revised the manuscript. All authors contributed to the article and approved the submitted version.

## Conflict of Interest

The authors declare that the research was conducted in the absence of any commercial or financial relationships that could be construed as a potential conflict of interest.

## Publisher's Note

All claims expressed in this article are solely those of the authors and do not necessarily represent those of their affiliated organizations, or those of the publisher, the editors and the reviewers. Any product that may be evaluated in this article, or claim that may be made by its manufacturer, is not guaranteed or endorsed by the publisher.
